# Outcomes and predictors of functioning, mental health, and health-related quality of life in adults born with very low birth weight: a prospective longitudinal cohort study

**DOI:** 10.1186/s12887-022-03676-6

**Published:** 2022-11-03

**Authors:** Arnt Erik Karlsen Wollum, Elias Kjølseth Berdal, Johanne Marie Iversen, Marit S. Indredavik, Kari Anne I. Evensen

**Affiliations:** 1 Faculty of Medicine and Health Sciences, Norwegian University of Science and Technology, Trondheim, Norway; 2 Department of Internal Medicine, Nordland Hospital Trust, Bodø, Norway; 3 Department of Clinical Medicine, UiT Arctic University of Norway, Tromsø, Norway; 4 Department of Clinical and Molecular Medicine, Norwegian University of Science and Technology, Trondheim, Norway; 5 Unit for Physiotherapy Services, Trondheim Municipality, Trondheim, Norway; 6 Children’s Clinic, St. Olavs Hospital, Trondheim University Hospital, Trondheim, Norway; 7 Department of Rehabilitation Science and Health Technology, Oslo Metropolitan University, Oslo, Norway

**Keywords:** General functioning, Health-related quality of life, Mental health, Preterm, Very low birth weight

## Abstract

**Background:**

Very low birth weight (VLBW: ≤1500 g) is associated with multiple short and long-term complications. This study aimed to examine outcomes and predictors of functioning, mental health, and health-related quality of life in adults born with VLBW.

**Methods:**

In this prospective longitudinal cohort study, 67 VLBW and 102 control participants were assessed using the Adult Self-Report of the Achenbach System of Empirically Based Assessment and Global Assessment of Functioning at 26 years, and the Hospital Anxiety and Depression Scale and Short Form-36 at 28 years of age. Associations between perinatal and childhood predictors and adult functioning were assessed using linear regression.

**Results:**

Compared with controls, the VLBW group had lower mean raw scores on the Function and Symptom subscales of the Global Assessment of Functioning at 26 years, a higher sum score of symptoms of anxiety and depression due to more depressive symptoms, and poorer mental health-related quality of life at 28 years. The mean group differences ranged from 0.42 to 0.99 SD. Within the VLBW group, lower birth weight and gestational age, a higher number of days with respiratory support and poorer motor function at 14 years were associated with a higher sum score of symptoms of anxiety and depression at 28 years. Days with respiratory support and motor function at 14 years were also predictive of Global Assessment of Functioning scores at 26 years, and mental health-related quality of life at 28 years. Poorer motor and cognitive function at five years were associated with poorer physical health-related quality of life at 28 years. Parental socioeconomic status was related to mental and physical health-related quality of life.

**Conclusion:**

In this study, VLBW adults reported poorer functioning and mental health-related quality of life, and more depressive symptoms than their term born peers. Days with respiratory support and adolescent motor function predicted most of the adult outcomes. This study explicates perinatal and developmental markers during childhood and adolescence which can be target points for interventions.

**Supplementary Information:**

The online version contains supplementary material available at 10.1186/s12887-022-03676-6.

**Background**.

Approximately 1.5% of children worldwide are born with a very low birth weight (VLBW) (birth weight ≤ 1500 g) [1]. Being born with VLBW is related to several short-term complications related to organ dysmaturation, such as respiratory problems, motor impairments, and neurodevelopmental difficulties [2–5]. Over the past decades, survival rates of VLBW infants have drastically increased [6], leading to an increasing population of adults born with VLBW. Studies have shown that VLBW in addition to perinatal morbidity also is a risk factor for several long-term complications lasting into adulthood [7–9]. Findings from individual participant meta-analyses have documented a higher prevalence of adult psychopathology, especially anxiety and depressive symptoms, attention deficit and autism spectrum disorder [10, 11] as well as more internalising behaviour [12] and lower ratings for their relationships with friends [13]. We have previously reported that VLBW adults have lower general functioning compared with controls [14]. A systematic review of the literature on health-related quality of life yields mixed findings and no conclusive evidence of differences between term born adults and those born very preterm or with VLBW [15].

A theoretical framework of mechanisms possibly underlying these adult outcomes may include maternal and foetal infections and perinatal inflammation causing white matter damage to the preterm brain [16]. Along with socioeconomic disadvantage, this is likely to contribute to adverse neurodevelopmental outcomes, including cerebral palsy (CP) and cognitive impairments, as well as psychopathology [16]. On this background, possible predictive factors for adult outcomes may be perinatal as well as factors important for functioning through childhood and adolescence, such as motor and cognitive skills. A recent review article summarising the evidence of 38 articles, showed that VLBW individuals struggle with poorer motor function into adulthood [17]. Furthermore, individual participant data meta-analysis of 13 studies showed that adult intelligence was lower among very preterm or VLBW compared with controls [18].

Only a few studies have identified predicting factors for general functioning, mental health, and health-related quality of life in preterm born or VLBW populations who reach adulthood. In a Swedish adolescent VLBW sample, lower birth weight and gestational age as well as mechanical ventilation increased overall morbidity and the need for hospital care after the neonatal period [19]. In the current sample assessed at 14 years, we found that lower birth weight, shorter gestation, and intraventricular haemorrhage were risk factors for psychiatric problems in the VLBW group [20]. Furthermore, increasing length of respiratory support and hospital stay in the neonatal period as well as motor problems in adolescence were associated with psychiatric symptoms at 26 years of age [21]. We also found associations between poor motor function at 23 years of age and mental health problems and lower health-related quality of life at the same age [22], and others have reported an association between childhood cognitive function and adult health-related quality of life [23]. A review article highlights decreasing gestational age and lower sociodemographic status as major predictors of adult psychopathology [24], whereas others have found neither perinatal factors nor childhood cognitive function to predict psychiatric disorders in adulthood [25].

In this study, we aimed to (1) assess differences in adaptive and general functioning, symptoms of depression and anxiety, and health-related quality of life between young adults born with VLBW and term born controls, and (2) identify perinatal and childhood factors that could predict these adult outcomes. We hypothesised that VLBW young adults had poorer adaptive and general functioning, more anxious and depressive symptoms and poorer health-related quality of life compared with the control group. We further hypothesised that birth weight and gestational age, perinatal morbidity and poor motor and cognitive childhood function would predict lower functioning, poorer mental health, and reduced health-related quality of life in young adulthood.

## Methods

### Study design

**Fig. 1:**
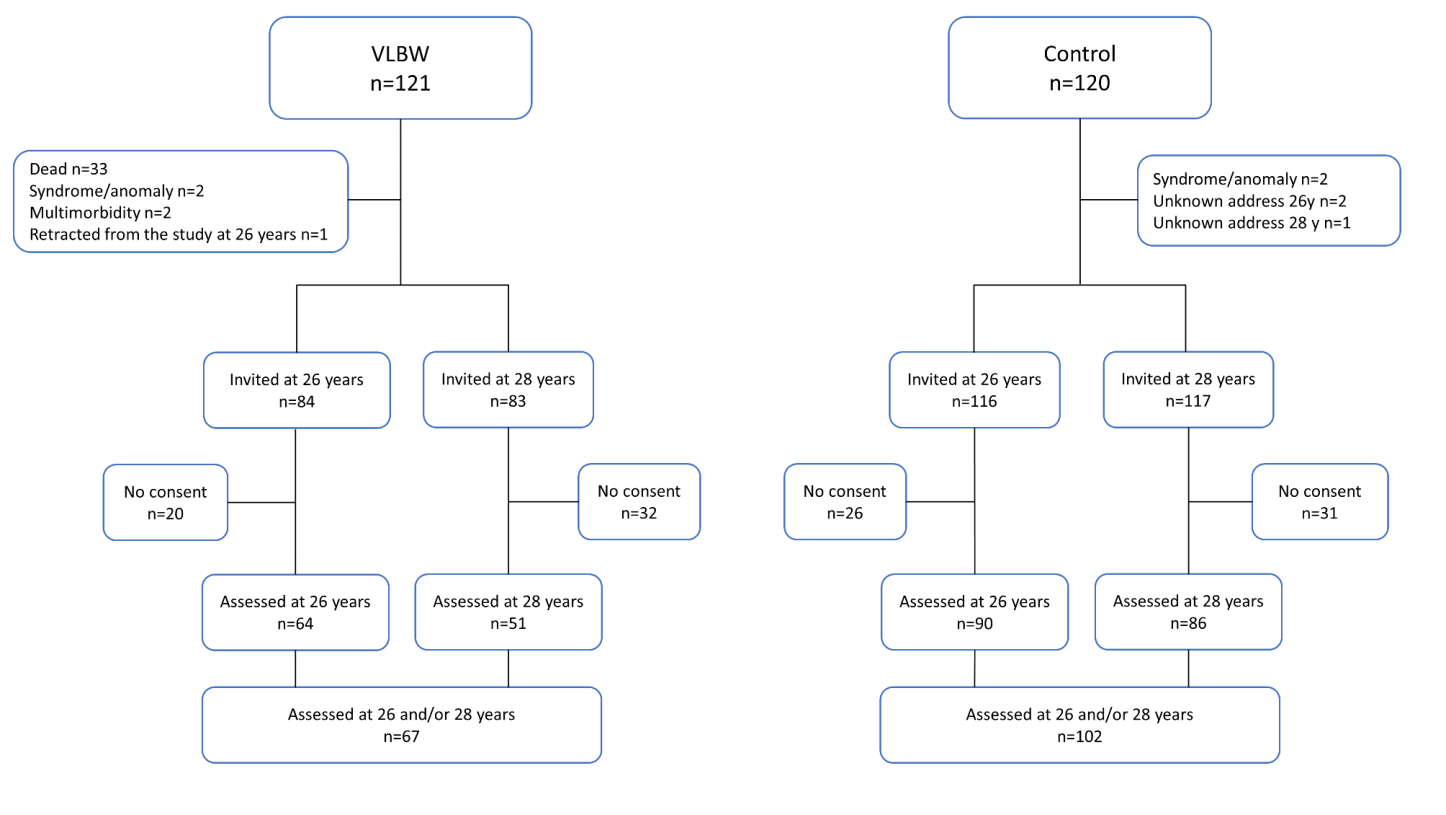
Flow of participants

This study is part of a longitudinal cohort study including a sample of preterm VLBW children and a term born normal birth weight control group [7]. All study participants were born between 1986 and 1988. Flow of participants is presented in Fig. 1. The VLBW participants were enrolled after birth and admission to the neonatal intensive care unit (NICU) of St. Olavs Hospital, Trondheim University Hospital, Norway, serving the counties of North and South Trøndelag as well as Møre and Romsdal. The control participants were enrolled during pregnancy as part of a multicentre study on consequences of intrauterine growth restriction [26], and born to mothers of a 10% random selection of all women residing in the Trondheim area. Both groups have been assessed at several time points during childhood, adolescence, and early adulthood. At 26 years, adaptive and general adult functioning was assessed. At age 28, anxious and depressive symptoms as well as health-related quality of life were assessed.

### Study sample

#### VLBW group

Of the 121 VLBW infants enrolled, 33 died in the neonatal period and two were excluded due to a diagnosis of a syndrome or malformation. Of the 86 eligible individuals, two were not invited due to multimorbidity and 20 did not consent at 26 years of age (23.8% of invited). One of the non-consenters at 26 years of age retracted from future follow-up. At 28 years of age, 83 were invited and 32 did not consent (38.6% of invited). Thus, data were available for 64 VLBW participants at 26 years and 51 VLBW participants at 28 years of age. In total, 67 VLBW participants were assessed at 26 and/or 28 years of age (Fig. 1).

#### Control group

The control group comprised 120 term born infants with birth weight ≥ 10th percentile for gestational age, corrected for sex and parity [26]. Of these, two children were excluded due to a diagnosis of syndrome or malformation, two were not invited due to unknown address and 26 did not consent to participation at 26 years (22.4% of invited). At 28 years, one was not invited due to unknown address and 31 did not consent to participation (26.5% of invited). Thus, data were available for 90 and 86 controls at 26 and 28 years of age, respectively. In total, 102 control participants were assessed at 26 and/or 28 years of age (Fig. 1).

#### Non-participants

Compared with participants, VLBW individuals who did not consent to participate at either 26 or 28 years were born to younger mothers (25.9 ± 3.0 vs. 28.3 ± 5.0 years, p = 0.019, Table S1). In the control group, those who did not consent had poorer fine motor scores at five years and lower IQ scores at five and 14 years of age compared with participants. There were no other differences in measured background characteristics between participants and those who did not consent to participate in either group.

### Background characteristics and predictors

Birth weight, gestational age, head circumference, prenatal maternal glucocorticoids, Apgar score at 5 min, days with respiratory support (ventilator or continuous positive airway pressure), days of stay in NICU or paediatric ward, presence of intraventricular haemorrhage (IVH) on neonatal cerebral ultrasound, bronchopulmonary dysplasia (BPD), necrotising enterocolitis (NEC), infant respiratory distress syndrome (IRDS), sepsis at birth and neonatal seizures were retrieved from hospital records.

To measure the socioeconomic status (SES) of the participants’ parents, the Hollingshead Two-Factor Index of Social Position [27] was used at the 14-year follow-up and supplemented at 19 years. The index is based on occupation and educational attainment, yielding a raw score range of 8 to 66, divided into five groups, where higher scores indicate increasing SES.

Motor function was assessed at one year of age by a test technician using the Psychomotor Development Index (PDI) of the Bayley Scales of Infant Development (BSID), yielding age-adjusted standard scores ranging from 50 to 150 with a mean of 100 and a standard deviation of 15, where lower scores indicates poorer psychomotor function [28]. At five years, motor function was assessed by a paediatrician using three subscales of the Peabody Developmental Motor Scales (PDMS), giving a maximal cumulative raw score of 86 for Fine Motor (eye-hand coordination), 66 for Balance and 116 for Locomotor subscales, where higher scores indicate better motor skills [29]. At 14 years, motor function was assessed by a physiotherapist using the Movement Assessment Battery for Children (MABC), giving a total score ranging from 0 to 40, where a higher score indicates poorer motor function [30]. The presence and severity of CP was assessed by two project paediatricians at 14 years of age and classified as hemiplegia, diplegia or quadriplegia.

Childhood cognitive function was assessed by a test technician using the Mental Development Index (MDI) of the BSID at one year of age [28], the Wechsler Preschool and Primary Scale of Intelligence-Revised (WPPSI-R) [31] at five years of age, and estimated by an experienced psychologist using two subscales (vocabulary and block design) of the Wechsler Intelligence Scale for Children-Third Edition (WISC-III) at 14 years of age [32]. As for the BSID PDI, the BSID MDI yields age-adjusted standard scores ranging between 50 and 150 with a mean of 100 and a standard deviation of 15 [28]. The WPPSI and WISC-III raw scores are converted into standardised IQ scores with a mean of 100 and a standard deviation of 15, where higher scores indicate higher intelligence [31, 32]. An estimated IQ score on the WISC-III more than two standard deviations below the mean in the control group (< 69) was defined as ‘Low estimated IQ’.

At one and five years of age, the assessors were blinded to neonatal data. At 14 years of age, the assessments were performed blinded to group affiliation.

### Outcome measures

#### Adult Self-Report

To measure adaptive functioning, including educational attainment and social aspects of friends and family, we used the Mean Adaptive Functioning Profile from the Adult Self-Report (ASR) of the Achenbach System of Empirically Based Assessment (ASEBA) [33]. This tool has been used in over 10.000 publications [34] and has shown good test-retest reliability, acceptable internal consistency and substantial long-term stability [33]. The Mean Adaptive Functioning Profile includes the five adaptive functioning scales “Friends” (0 to 12), “Spouse/Partner” (-8 to + 8), “Family” (0–2), “Job” (-10 to + 4) and “Education” (-4 to + 6). A Mean Adaptive score is computed using the T-scores from these adaptive functioning scales, divided by the number of completed scales. A higher Mean Adaptive score indicates better adaptive functioning [33]. Due to the nature of the assessment tool, results for Spouse/Partner were only available for 34 VLBW and 48 controls, 49 and 79 for Job, and 17 and 34 for Education, respectively.

#### Global Assessment of Functioning

General functioning was assessed by the Global Assessment of Functioning (GAF), an interview tool for assessing psychiatric symptoms as well as social and occupational level [35]. The GAF yields a continuous total score of 0-100, where higher scores indicate better outcomes. It has been proven both reliable and valid in multiple studies, although some findings indicate the need for an experienced evaluator [35]. In 1998, Karterud et al. [36] constructed a modified version of the GAF, split into a Function and a Symptom score, proven to be highly generalisable [35], which we applied in our study. The Function scale considers coping in everyday and social life, and participation in work and education. The Symptom scale comprises cognition, judgement, demeanour, and mood, and how psychiatric symptoms affect these traits. The GAF was conducted by an experienced clinician blinded to group affiliation of the participants. Mean GAF scores were previously published in a slightly smaller VLBW sample by Lærum et al. [14].

#### Hospital Anxiety and Depression Scale

To measure health aspects of anxiety and depression, we used a Norwegian translation of the Hospital Anxiety and Depression Scale (HADS) [37]. This is a widely used and well-validated [38–41] self-report questionnaire comprising a 14-item scale, where seven items relate to anxiety and seven relate to depression. Each item gives a score ranging from 0 to 3, resulting in a total score between 0 and 42, where 0 is no symptoms and 42 is the highest attainable symptom load. The summary score (HADS Total) can be subdivided into two categories: HADS Depression and HADS Anxiety score, each ranging from 0 to 21 points [42].

#### Short Form 36 Health Survey (SF-36)

To assess health-related quality of life we used the Short Form 36 Health Survey (SF-36). The SF-36 has been validated for use in a wide array of studies, both internationally and in Norwegian populations [43–45]. The questionnaire consists of 36 questions, intended to assess a persons’ physical and mental health aspects of quality of life. The survey results comprise eight domains: Physical Functioning, role limitations due to physical problems (Role-Physical), Bodily Pain, General Health, Vitality, Social Functioning, role limitations due to emotional problems (Role-Emotional) and Mental Health, and two composite summaries of physical and mental health. The raw scores are converted into percentages, ranging from 0 to 100. Lower percentages indicate poorer health outcome and a lower functioning level. The Physical Component Summary is mainly built upon the three domains of Physical Functioning, Role-Physical and Bodily Pain, while the Mental Component Summary mainly consists of the domains of Social Functioning, Role-Emotional and Mental Health. The component summaries are given as T-scores, based on an average of 50 points and a standard deviation (SD) of 10 points [45].

### Ethics

The study was conducted in accordance with the Helsinki Declaration and approved by the Regional Ethics Committee (REK) in Central Norway (78 − 00 May 2000 and 2013/636). The parents gave written informed consent on behalf of their children at birth and childhood assessments. The adult participants gave their written informed consent.

### Statistical analysis

Data were analysed using IBM®SPSS®Statistics version 27. Statistical significance was set at two-sided p-values below 0.05. Group differences were analysed with chi-square statistics for categorical data, Student’s t-test for continuous and normally distributed data and Mann- Whitney U test for ordinal data. Normality was assessed by visual inspection of histograms and Q-Q plots of the residuals. Due to slight deviations from normality for the outcome measures, we used bootstrapping with B = 2000 bootstrap samples and bias-corrected and accelerated (BCa) method. Mann-Whitney U test was used for variables where we did not obtain a p-value by bootstrapping. We calculated effect sizes in SD units, and magnitude of the difference between groups were interpreted as small (0.2), medium (0.5) or large (0.8) [46]. We carried out sensitivity analyses excluding participants with CP and low estimated IQ. Sex and parental SES were included as possible confounders. A univariable general linear model was applied to examine the effect of predictors on adult outcomes within the VLBW group. For all analyses, we used the available data, and we did not impute missing values.

## Results

### Background characteristics

Background characteristics of the participants are presented in Table 1. Mothers of VLBW participants were younger than mothers of controls, but parental SES did not differ between the groups. As expected, birth weight, gestational age, and head circumference at birth were lower in the VLBW than in the control group. The VLBW group had lower Apgar score at 5 min and poorer childhood motor and cognitive function (Table 1).

**Table 1:** Background characteristics of very low birth weight participants and term born controls

	VLBW			Control			
	n	Mean	(SD)	n	Mean	(SD)	*p*
**Maternal factors**							
Maternal age at birth, years	66	28.3	(5.0)	98	30.3	(4.4)	0.006
Parental SES	56	3.4	(1.3)	80	3.8	(1.1)	0.179
Maternal glucocorticoids, n (%)	65	34	(52.3)	-	-		-
**Perinatal factors**							
Male, n (%)	67	35	(52.2)	102	42	(41.2)	
Birth weight, g	67	1184	(254)	102	3719	(464)	< 0.001
Gestational age, weeks	67	28.9	(2.6)	102	39.8	(1.2)	< 0.001
Head circumference at birth, cm	53	27.0	(2.4)	97	35.4	(1.1)	< 0.001
Apgar at 5 min	65	8.3	(1.7)	97	9.8	(1.0)	< 0.001
Days with respiratory support	66	5.5	(11.6)	-	-	-	-
Days in NICU, median (IQR)	56	61.0	(49.3–87.8)	-	-	-	-
IVH, n (%)	56	6	(10.7)	-	-	-	-
BPD, n (%)	64	14	(21.9)	-	-	-	-
IRDS, n (%)	65	34	(52.3)	-	-	-	-
NEC, n (%)	56	2	(3.6)	-	-	-	-
Sepsis at birth, n (%)	56	7	(12.5)	-	-	-	-
Neonatal seizures, n (%)	67	5	(7.5)	-	-	-	-
**Motor function**							
BSID PDI 1y	24	100.0	(18.0)	86	108.2	(11.7)	0.044
PDMS Fine Motor 5y	25	79.2	(5.7)	82	80.9	(3.3)	0.175
PDMS Balance 5y	24	57.6	(4.6)	82	59.2	(4.4)	0.130
PDMS Locomotor 5y	24	101.1	(10.9)	82	105.9	(5.6)	0.047
MABC Total 14y	45	11.3	(6.2)	73	6.2	(4.1)	< 0.001
Cerebral palsy, n (%)	67	4	(6.0)	102	0	0	0.023
**Cognitive function**							
BSID MDI 1y	24	88.2	(15.9)	86	120.8	(10.5)	< 0.001
WPPSI-R 5y	19	94.8	(17.1)	87	107.3	(12.3)	< 0.001
WISC-III 14y	49	87.6	(19.8)	74	98.3	(14.1)	0.002
Low estimated IQ, n (%)	49	9	(18.4)	74	2	(2.7)	0.004

BPD: Bronchopulmonary dysplasia, BSID: Bayley Scales of Infant Development, IQ: Intelligence quotient, IQR: Interquartile range, IRDS: Infant respiratory distress syndrome, IVH: Intraventricular haemorrhage, NEC: Necrotizing enterocolitis, NICU: Neonatal intensive care unit, PDI: Psychomotor Development Index, PDMS: Peabody Developmental Motor Scales, MABC: Movement Assessment Battery for Children, MDI: Mental Development Index, SD: Standard deviation, SES: Socioeconomic status, VLBW: Very low birth weight, WPPSI-R: Wechsler Preschool and Primary Scale of Intelligence – Revised, WISC-III: Wechsler Intelligence Scale for Children – Third edition. p-values for differences in continuous variables based on Student’s t-test, except for Parental SES and Days in NICU, where p-values are based on Mann-Whitney U test. p-values for differences in proportions based on Pearson’s chi square test

### Adult outcomes

Table 2 shows the results of ASR, GAF, HADS and SF-36. At 26 years of age, the VLBW group had lower scores for the adaptive functioning scale Job, with a mean difference of 0.58 SD units compared with the control group but Mean Adaptive score did not differ between the two groups. The VLBW group scored significantly lower on both GAF subscales with mean differences of 0.68 SD for the Symptom subscale and 0.99 SD for the Function subscale, indicating medium to large effect sizes.

At 28 years of age, HADS Depression and HADS Total scores were higher in the VLBW group than in the control group, with a mean difference of 0.54 and 0.42 SD, respectively. VLBW individuals also scored significantly lower on several SF-36 domains, with mean differences ranging from 0.45 to 0.72 SD. The Mental Component Summary was lower, but the Physical Component Summary did not differ between the groups.

**Table 2:** Adult outcomes of very low birth weight participants compared with term born controls

	VLBW			Control			SD difference	
	n	Mean	(SD)	n	Mean	(SD)		*p*
**ASR Mean Adaptive Profile**								
Friends	60	9.2	(2.7)	88	9.9	(2.1)	0.33	0.070
Spouse/Partner	34	6.0	(2.3)	48	5.7	(2.9)	0.10	0.929^a^
Family	59	1.4	(0.4)	88	1.5	(0.4)	0.25	0.507
Job	49	2.3	(1.6)	79	3.0	(1.2)	0.58	0.024
Education	17	3.5	(1.9)	34	4.3	(1.9)	0.42	0.162
Mean Adaptive	60	48.3	(9.3)	88	50.9	(7.9)	0.33	0.059
**GAF**								
GAF Function	52	78.8	(17.3)	81	87.4	(8.7)	0.99	0.006
GAF Symptom	52	79.4	(15.5)	81	86.4	(10.3)	0.68	0.008
**HADS**								
HADS Anxiety	48	5.3	(4.1)	85	4.4	(3.6)	0.25	0.230
HADS Depression	48	3.5	(3.2)	85	2.0	(2.8)	0.54	0.010
HADS Total	48	8.8	(6.7)	85	6.4	(5.7)	0.42	0.040
**SF-36**								
Physical Functioning	51	92.3	(11.6)	86	96.1	(7.6)	0.50	0.052
Role-Physical	51	77.5	(34.7)	86	89.8	(26.4)	0.47	0.081
Bodily Pain	51	72.4	(25.7)	86	79.6	(21.8)	0.33	0.230
General Health	51	74.3	(23.3)	86	81.7	(16.0)	0.46	0.109
Vitality	51	48.5	(19.3)	86	56.5	(17.9)	0.45	0.023
Social Functioning	51	83.8	(22.3)	86	93.2	(15.2)	0.70	0.024
Role-Emotional	51	77.8	(33.8)	86	92.6	(21.3)	0.69	0.017
Mental Health	51	71.1	(20.5)	86	80.8	(13.4)	0.72	0.005
Physical Component Summary	51	53.1	(8.2)	86	54.8	(6.7)	0.25	0.381
Mental Component Summary	51	46.2	(12.0)	86	51.8	(8.4)	0.67	0.008

ASR: Adult Self-Report, GAF: Global Assessment of Functioning, HADS: Hospital Anxiety and Depression Scale, SD: Standard deviation, SF-36: Short Form 36 Health Survey, VLBW: Very low birth weight. All scores are raw scores, except the Physical and Mental Component Summaries, which are T-scores based on an average of 50 points and a standard deviation of 10 points. The Mean Adaptive score is based on average T-scores from the five adaptive scales. p-values based on bias-corrected and accelerated bootstrap (BCa). ^a^ p-value based on Mann-Whitney U test as we did not obtain a p-value based on bootstrapping.

**Table 3:** Predictors of adult outcomes in very low birth weight participants

	ASR Mean Adaptive			GAF Function			GAF Symptom			HADS Total			SF-36 Mental			SF-36 Physical		
	B	(95% CI)	*p*	B	(95% CI)	*p*	B	(95% CI)	*p*	B	(95% CI)	*p*	B	(95% CI)	*p*	B	(95% CI)	*p*
**Maternal factors**																		
Maternal glucocorticoids	0.1	(-2.8, 2.9)	0.969	3.7	(-5.8, 13.8)	0.447	0.8	(-8.3, 9.4)	0.856	-1.8	(-5.7, 2.2)	0.370	1.3	(-5.8, 8.2)	0.699	-0.4	(-5.2, 4.2)	0.859
Parental SES	-0.5	(-1.7,0.7)	0.421	-1.7	(-6.0, 2.7)	0.504	-1.7	(-5.7, 2.6)	0.471	0.9	(-0.3, 2.1)	0.138	-2.5	(-4.9, -0.4)	0.019	2.1	(0.4, 4.2)	0.023
**Perinatal Factors**																		
Birthweight (pr.100 g)	0.3	(-0.2, 0.8)	0.282	2.5	(-0.1, 4.5)	0.048	1.1	(-0.8, 3.0)	0.264	-1.0	(-1.7, -0.3)	0.011	1.0	(-0.8, 2.4)	0.237	-0.4	(-1.4, 0.6)	0.401
Gestational age (weeks)	0.5	(-0.01, 0.9)	0.058	0.6	(-1.7, 2.9)	0.577	0.6	(-1.5, 2.8)	0.534	-0.6	(-1.0, 0.1)	0.040	0.4	(-0.9, 1.4)	0.477	-0.4	(-1.2, 0.4)	0.335
Apgar at 5 min	0.1	(-0.8, 0.9)	0.853	1.3	(-1.7, 4.8)	0.425	0.7	(-2.3, 4.3)	0.679	-0.7	(-1.9, 0.3)	0.191	0.6	(-1.6, 2.6)	0.548	-0.6	(-1.9, 0.8)	0.344
Days with respiratory support	-0.1	(-0.2, -0.03)	0.102	-0.5	(-0.9, 0.01)	0.019	-0.5	(-0.9, 0.1)	0.020	0.2	(0.1, 0.6)	0.013	-0.3	(-0.7, -0.2)	0.024	0.1	(-0.1, 0.2)	0.233
Days in NICU	0.01	(-0.04, 0.02)	0.592	-0.1	(-0.3, 0.03)	0.513	-0.01	(-0.2, 0.04)	0.778	0.02	(-0.06, 0.1)	0.362	-0.01	(-0.2, 0.03)	0.870	-0.02	(-0.1, 0.1)	0.335
IVH	0.3	(-1.6, 5.5)	0.674	-6.8	(-11.6, 14.2)	0.066	-7.8	(-11.8, 13.3)	0.030	3.1	(-1.5, 7.9)	0.056	-4.8	(-15.5, 6.7)	0.119	2.0	(-5.7, 4.2)	0.276
BPD	-2.3	(-5.6, 1.0)	0.189	-10.0	(-22.4, 2.5)	0.128	-7.9	(-20.6, 4.6)	0.228	3.8	(-0.2, 7.9)	0.062	-2.6	(-10.0, 4.2)	0.501	0.6	(-4.4, 6.2)	0.823
Sepsis at birth	4.2	(-0.1, 8.2)	0.040	-0.5	(-31.4, 16.3)	0.969	8.3	(1.2, 14.7)	0.025	-2.1	(-5.7, 1.1)	0.202	7.1	(0.5, 13.5)	0.030	-2.2	(-9.1, 4.8)	0.472
**Motor Function**																		
BSID PDI 1y	0.03	(-0.1, 0.2)	0.787	-0.02	(-0.3, 0.5)	0.848	-0.1	(-0.3, 0.4)	0.590	-0.1	(-0.4, 0.04)	0.204	0.1	(-0.1, 0.5)	0.653	0.1	(-0.2, 0.3)	0.196
PDMS Eye-hand 5y	0.1	(-0.3, 0.6)	0.635	1.1	(-0.2, 2.5)	0.305	0.8	(-0.7, 2.2)	0.463	-0.4	(-0.8, 0.2)	0.122	0.3	(-0.6, 1.0)	0.608	0.6	(0.2, 1.1)	0.008
PDMS Balance 5y	0.4	(-0.6, 0.9)	0.450	0.8	(-2.0, 3.4)	0.659	0.5	(-2.3, 3.1)	0.794	-0.3	(-1.1, 1.2)	0.459	0.2	(-1.2, 1.1)	0.735	0.7	(-0.1, 1.2)	0.026
PDMS Locomotor 5y	0.1	(-0.2, 0.4)	0.558	0.5	(-0.4, 1.3)	0.390	0.3	(-0.6, 1.1)	0.627	-0.2	(-0.4, 0.3)	0.268	0.1	(-0.3, 0.5)	0.592	0.3	(-0.1, 0.5)	0.045
MABC Total 14y	-0.1	(-0.3, 0.3)	0.650	-0.7	(-1.5, 0.4)	0.080	-0.8	(-1.5, 0.4)	0.042	0.4	(0.1, 0.6)	0.003	-0.7	(-1.0, -0.1)	0.002	-0.2	(-0.5, 0.1)	0.212
Cerebral palsy	-2.6	(-16.7, 9.4)	0.686	-28.1	(-61.8, 13.4)	0.169	-12.1	(-38.6, 3.5)	0.291	2.0	(-7.5, 8.9)	0.658	1.3	(-10.8, 16.1)	0.846	-1.2	(-11.9, 15.5)	0.894
**Cognitive Function**																		
BSID MDI 1y	0.02	(-0.2, 0.3)	0.863	-0.04	(-0.3, 0.3)	0.679	-0.2	(-0.4, 0.1)	0.120	0.1	(-0.1, 0.4)	0.513	-0.2	(-0.7, 0.1)	0.213	0.1	(-0.2, 0.3)	0.372
WPPSI 5y	-0.02	(-0.2, 0.1)	0.800	0.01	(-0.5, 0.3)	0.971	-0.03	(-0.5, 0.2)	0.776	-0.02	(-0.1, 0.1)	0.773	-0.1	(-0.3, 0.2)	0.281	0.2	(0.1, 0.4)	0.007
WISC-III 14y	-0.03	(-0.1, 0.04)	0.411	0.4	(0.1, 0.7)	0.053	0.1	(-0.05, 0.4)	0.186	-0.1	(-0.1, 0.04)	0.198	0.1	(-0.1, 0.3)	0.499	0.1	(-0.02, 0.2)	0.057

ASR: Adult Self-Report, B: Unstandardised beta; BPD: Bronchopulmonary Dysplasia, BSID: Bayley Scales of Infant Development, IVH: Intraventricular Haemorrhage, MABC: Movement Assessment Battery for Children, MDI: Mental Development Index, PDI: Psychomotor Development Index, PDMS: Peabody Developmental Motor Scales, SES: Socioeconomic status, SF-36: Short Form 36 Health Survey, WISC-III: Wechsler Intelligence Scale for Children – Third edition, WPPSI-R: Wechsler Preschool and Primary Scale of Intelligence – Revised. Linear regression using ASR Mean Adaptive score, GAF Function and Symptom scores, HADS Total score and SF-36 Physical and Mental Component Summaries as dependent variables, and maternal and perinatal factors as well as motor and cognitive function as independent variables. Confidence intervals and p-values are based on bias-corrected and accelerated bootstrap (BCa).

### Sensitivity analyses

When we excluded participants with CP (four VLBW participants), the results were essentially unchanged (data not shown). When we excluded participants with low estimated IQ (nine VLBW and two control participants), most outcomes improved slightly (2–7%) in the VLBW group causing smaller differences between the groups, and differences in GAF Function and SF-36 Mental Health scores were no longer significant.

### Confounders

When we adjusted the adult outcomes for sex and parental SES separately, the results were essentially unchanged (data not shown).

### Predictors of adult outcomes in the VLBW group

Table 3 shows the results from linear regression using ASR Mean Adaptive score, GAF Function and Symptom scores, HADS Total and SF-36 Physical and Mental Component Summaries as dependent variables, and maternal and perinatal factors as well as motor and cognitive function as independent variables. A higher parental SES was related to a lower SF-36 Mental but a higher Physical Component Summary. Higher birth weight was associated with higher GAF Function and lower HADS Total score. Higher gestational age was also associated with lower HADS Total score. A higher number of days with respiratory support after birth was associated with lower GAF Function and Symptom scores, higher HADS Total score and lower SF-36 Mental Component Summary. Presence of IVH was associated with a lower GAF Symptom score. Sepsis at birth was associated with higher ASR Mean Adaptive score, GAF Symptom score and SF-36 Mental Component Summary. Better motor and cognitive function at 5 years of age were associated with a higher SF-36 Physical Component Summary. Poorer motor function at 14 years were associated with lower GAF Symptom score, higher HADS Total score and lower SF-36 Mental Component Summary. The absolute values of the standardised regression coefficients for the predictors showing significant associations with the outcomes ranged from 0.19 to 0.58 (Table S2).

## Discussion

### Main findings

In this study, VLBW adults had poorer general functioning, more depressive symptoms, and a poorer mental health-related quality of life than term born controls with effect sizes ranging from medium to large in magnitude. Within the VLBW group, we found that lower birth weight and gestational age, a higher number of days with respiratory support and poorer motor function at 14 years were associated with a higher sum score of symptoms of anxiety and depression at 28 years. Days with respiratory support and motor function at 14 years were also predictive of Global Assessment of Functioning scores at 26 years, and mental health-related quality of life at 28 years. Poorer motor and cognitive function at five years were associated with poorer physical health-related quality of life at 28 years. Parental socioeconomic status was related to mental and physical health-related quality of life.

### Strengths and limitations

A major strength of this study is the longitudinal prospective design with a long follow-up time of a VLBW and a control group from birth and up to 28 years. As our study groups included individuals born preterm with VLBW defined by a cut-off of 1500 g and individuals born at term with birth weight at or above the 10th percentile, we are missing the full range of birth weights in both groups. This may extrapolate our results, making the contrasts larger than if we had also included individuals representing the whole birth weight spectrum. Loss to follow-up during such a long time period is inevitable [47], and was also present in our study. This limits our statistical power to detect differences, leading to a risk of type 2 errors. The sample size was especially limited when we excluded participants with low estimated IQ and adjusted for parental SES. On the other hand, multiple analyses may lead to a possible inflation of type 1 errors. However, as our sample size was limited, we were only able to demonstrate significant differences for medium to large effects. Further, loss to follow-up may lead to selection bias and impact the generalisability of our results. The VLBW who did not consent were born to younger mothers, which probably did not notably affect the results as parental SES did not differ. Non-consenting controls had lower IQ scores than participating controls, however there were no differences in the proportions with low estimated IQ. Thus, we consider our participants to be representative of the initial sample.

Another strength of the study is the use of well-validated and widely used tools to assess motor skills [28–30], cognitive function [32] and adult outcomes [33, 36, 42, 45]. Objective assessments of the participants’ motor skills, cognitive function, and general functioning were performed by experienced professionals blinded to neonatal history in childhood and group affiliation in adolescence and adulthood. In addition, we included self-assessment tools, enabling the participants to share their own perceptions of their lives. However, self-reports are susceptible to response bias, such as social desirability bias, and poorer cognitive function could possibly affect the ability to answer the questionnaires correctly. Therefore, we also performed sensitivity analyses, excluding participants with CP and/or low estimated IQ.

### Adult outcomes

The present study provides further evidence for the struggles of VLBW individuals. Our finding of a poorer work situation is supported by several other studies showing that VLBW individuals have lower rates of higher education and university grades [48–50], also found in the current sample at 26 years, with higher rates of unemployment and welfare dependency [14]. We found no group differences in the other adaptive scales, but poorer social functioning and coping abilities in everyday living represented by the GAF Function score. Other studies have also reported that VLBW adults have fewer friends and are more likely to be living with their parents at 19–22 years of age [48]. However, some studies state that VLBW adults show the same level of involvement in social activities [48], and no differences in scales of autonomy and social development [51].

In our study, the VLBW group scored significantly lower on the GAF Symptom subscale and higher on HADS Depression and Total symptoms compared with controls. Assessed by psychiatric interview at 26 years of age, VLBW participants had more overall psychiatric diagnoses, anxiety and mood disorders, and more self-perceived psychiatric problems assessed by ASEBA ASR [14, 21]. In the present study the higher sum score of symptoms of anxiety and depression was mainly due to higher mean depression score in the VLBW group compared with the control group.

The VLBW participants had a reduced health-related quality of life as indicated by a lower score in several domains of the SF-36 as well as for the Mental Component Summary. Previous findings of health-related quality of life in this cohort found few differences in SF-36 scores at 20 years [52], similar as in other cohorts assessed at the same age [53]. Even though some studies have reported no differences between VLBW and term born adults also at 19–22 and 22–23 years [48, 50], we have previously reported that differences were larger at 23 years of age [22], consistent with other studies of preterm born adults [54, 55]. This may indicate increasing difficulties in the transition to adulthood.

When we excluded participants with low estimated IQ, smaller group differences were observed, which was not the case when we excluded participants with CP. Thus, some of the differences seemed to be explained by lower cognitive function but independent of major neuromotor disability like CP. It is reasonable to assume that VLBW adults with low cognitive function may experience more educational challenges, which could lead to a poorer work situation, and in turn higher levels of stress and thus lower general functioning and health-related quality of life. When we adjusted for sex and parental SES, the results were essentially unchanged, indicating that the reported differences were not explained by these factors.

### Predictors of adult outcomes

Of the possible predictors examined in this study, perinatal factors such as lower birth weight and gestational age, and a higher number of days with respiratory support, were related to a higher sum score of anxious and depressive symptoms. In the proposed theoretical framework, a dynamic interaction between biological and environmental factors is plausible. In line with this, we found that parental SES was related to health-related quality of life. A few other studies have assessed associations between perinatal factors and adult mental health. Hack [24] reported in a review article that the two major predictors of adult psychopathology were gestational age and family socioeconomic status.

In a previous study of the present sample, Lærum et al. [21] found that increasing number of days with respiratory support and motor problems at 14 years were predictive for more psychiatric symptoms, and we found that these two factors were associated with several of the outcomes measured at 26 and/or 28 years. We have previously also reported associations between poor motor function at 23 years of age and mental health problems and lower health-related quality of life at the same age [22]. Magnetic resonance imaging studies have shown that VLBW children have in general more grey and white matter pathology [56], which are found to be associated with both cognitive and motor function [7]. A higher prevalence of motor problems at 14 years might indicate poorer developed cortices [57] as well as white matter tracts [58], and can thus be a marker for affected brain development which may also involve increased susceptibility to psychiatric morbidity. In addition, poor motor function in adolescence may impact participation and subsequently mental health and health-related quality of life.

Like Breeman et al. 2017 [23] who reported that lower health-related quality of life was related to cognitive impairment in adulthood, we found cognitive function at five years to be associated with physical health-related quality of life. Further, we found cognitive function at 14 years of age to be associated with the GAF Function subscale. It is a reasonable assumption that the weak association between adolescent cognitive function and adult general functioning could be explained by lower educational and occupational attainment.

### Clinical implications

This study establishes possible target points to prevent future struggle in the adult life of VLBW individuals. The observed associations of perinatal and childhood factors with adult functioning suggest that being born VLBW has long-term consequences. Even though most risk factors are related to poor organ and brain maturation in particular, knowledge of long-term outcome may guide supporting measures. The proposed theoretical framework suggests that affected brain development, especially succeeding white matter damage, lay the basis for developmental trajectories of skills and function. The smaller and sicker these individuals are at birth and in the neonatal period, the higher the chances are for adult mental health problems, as well as reduced general functioning and health-related quality of life. This study reinforces the knowledge about VLBW as a risk factor for later adverse mental health, social, adaptive, and quality of life outcomes. Further, it raises awareness regarding possibly modifiable neonatal and childhood factors important to the outcomes in VLBW adults. By identifying possible predictors of adverse outcomes, suitable interventions could be applied to the VLBW individuals at the highest risk. Thus, this study underlines the need for a thorough evaluation, follow-up, treatment, and adaptive support of VLBW children.

## Conclusion

Young adults born with VLBW had poorer general functioning, more depressive symptoms, and poorer mental health-related quality of life compared to term born peers. Number of days with respiratory support and adolescent motor function predicted most of the adult outcomes. This study explicates perinatal and developmental markers during childhood and adolescence which can be target points for interventions.

## Electronic supplementary material

Below is the link to the electronic supplementary material.

Supplementary Material 1

Supplementary Material 2

## Data Availability

The datasets generated and/or analysed during the current study are not publicly available because permission has not been applied for from neither the participants nor the Ethical Committee but might be available from the corresponding author on reasonable request.

## List of abbreviations

**ASEBA**: Achenbach System of Empirically Based Assessment
**ASR**: Adult Self-Report
**BSID**: Bayley Scales of Infant Development
**GAF**: Global Assessment of Functioning
**IVH**: Intraventricular haemorrhage
**HADS**: Hospital Anxiety and Depression Scale
**MABC**: Movement Assessment Battery for Children
**MDI**: Mental Development Index
**NEC**: Necrotizing enterocolitis
**NICU**: Neonatal Intensive Care Unit
**PDI**: Psychomotor Development Index
**PDMS**: Peabody Developmental Motor Scales
**SES**: Socioeconomic status
**SF-36**: Short-Form 36 Health Survey
**SGA**: Small for gestational age
**VLBW**: Very low birth weight
**WISC-III**: Wechsler Intelligence Scale for Children – Third edition
**WPPSI-R**: Wechsler Preschool and Primary Scale of Intelligence – Revised
