# Supplementary material for: Outcomes and predictors of functioning, mental health, and health-related quality of life in adults born with very low birth weight: a prospective longitudinal cohort study
Source: BMC Pediatr. 2022 Nov 3;22:628. doi: 10.1186/s12887-022-03676-6 (PMC9632018; doi:10.1186/s12887-022-03676-6)
Supplement: Supplementary file 1 — Supplementary Material 1 [file 12887_2022_3676_MOESM1_ESM.docx]

**Table S1.** Background characteristics of participants and individuals who did not consent to participation in the VLBW and control group.

|  | **VLBW** | | | | | | | **Control** | | | | | | |
| --- | --- | --- | --- | --- | --- | --- | --- | --- | --- | --- | --- | --- | --- | --- |
|  |  | Consent | |  | No consent | |  |  | Consent | |  | No consent | |  |
|  | n | Mean | (SD) | n | Mean | (SD) | *p* | n | Mean | (SD) | n | Mean | (SD) | *p* |
| **Maternal factors** |  |  |  |  |  |  |  |  |  |  |  |  |  |  |
| Maternal age at birth, years | 66 | 28.3 | (5.0) | 16 | 25.9 | (3.0) | 0.019 | 98 | 30.3 | (4.4) | 16 | 29.4 | (4.1) | 0.428 |
| Parental SES | 56 | 3.4 | (1.3) | 6 | 2.7 | (1.2) | 0.164 | 80 | 3.8 | (1.1) | 3 | 3.7 | (1.5) | 1.000 |
| Maternal glucocorticoids, n (%) | 65 | 34 | (52.3) | 15 | 8 | (53.3) | 0.943 |  | - | - | - | - | - | - |
| **Perinatal factors** |  |  |  |  |  |  |  |  |  |  |  |  |  |  |
| Male, n (%) | 67 | 35 | (52.2) | 16 | 12 | (75.0) | 0.099 | 102 | 42 | (41.2) | 16 | 9 | (56.3) | 0.258 |
| Birth weight, g | 67 | 1184 | (254) | 16 | 1123 | (178) | 0.386 | 102 | 3719 | (464) | 16 | 3591 | (311) | 0.291 |
| Gestational age, weeks | 67 | 28.9 | (2.6) | 16 | 28.3 | (2.4) | 0.472 | 102 | 39.8 | (1.2) | 16 | 39.3 | (1.4) | 0.182 |
| Head circumference at birth, cm | 53 | 27.0 | (2.4) | 11 | 26.9 | (1.8) | 0.933 | 97 | 35.4 | (1.1) | 16 | 35.1 | (1.2) | 0.316 |
| Apgar at 5 min | 65 | 8.3 | (1.7) | 15 | 8.8 | (1.0) | 0.229 | 97 | 9.8 | (1.9) | 16 | 9.9 | (0.3) | 0.804 |
| Days with respiratory support | 66 | 5.5 | (11.6) | 16 | 6.6 | (11.8) | 0.741 | - | - | - | - | - | - | - |
| Days in NICU, median (IQR) | 56 | 61.0 | (49.3-87.8) | 12 | 61.5 | (49.3-63.8) | 0.421 | - | - | - | - | - | - | - |
| IVH, n (%) | 56 | 6 | (10.7) | 12 | 1 | (8.3) | 1.000 | - | - | - | - | - | - | - |
| BPD, n (%) | 64 | 14 | (21.9) | 16 | 7 | (43.8) | 0.110 | - | - | - | - | - | - | - |
| IRDS, n (%) | 65 | 34 | (52.3) | 16 | 12 | (75.0) | 0.101 | - | - | - | - | - | - | - |
| NEC, n (%) | 56 | 2 | (3.6) | 12 | 0 | (0) | 1.000 | - | - | - | - | - | - | - |
| Sepsis at birth, n (%) | 56 | 7 | (12.5) | 12 | 3 | (25.0) | 0.365 | - | - | - | - | - | - | - |
| Neonatal seizures, n (%) | 67 | 5 | (7.5) | 16 | 1 | (6.3) | 1.000 | - | - | - | - | - | - | - |
| **Motor function** |  |  |  |  |  |  |  |  |  |  |  |  |  |  |
| BSID PDI 1y | 24 | 100.0 | (17.9) | 3 | 79.7 | (27.1) | 0.089 | 86 | 108.2 | (11.7) | 11 | 114.6 | (8.9) | 0.086 |
| PDMS Fine Motor 5y | 25 | 79.2 | (5.7) | 3 | 78.3 | (4.0) | 0.794 | 82 | 80.9 | (3.3) | 14 | 78.2 | (3.9) | 0.008 |
| PDMS Balance 5y | 24 | 57.6 | (4.6) | 3 | 56.3 | (4.2) | 0.658 | 82 | 59.2 | (4.4) | 14 | 58.8 | (4.8) | 0.779 |
| PDMS Locomotor 5y | 24 | 101.1 | (10.9) | 3 | 106.7 | (6.7) | 0.401 | 82 | 105.9 | (5.6) | 14 | 102.2 | (9.0) | 0.158 |
| MABC Total 14y | 45 | 11.3 | (8.1) | 11 | 9.8 | (7.0) | 0.565 | 73 | 6.2 | (4.1) | 7 | 5.6 | (4.8) | 0.745 |
| Cerebral palsy, n (%) | 67 | 4 | (6.0) | 16 | 1 | (6.3) | 1.000 | 102 | 0 | (0) | 16 | 0 | (0) | - |
| **Cognitive function** |  |  |  |  |  |  |  |  |  |  |  |  |  |  |
| BSID MDI 1y | 24 | 88.2 | (15.9) | 3 | 79.0 | (27.6) | 0.390 | 86 | 120.8 | (10.5) | 11 | 125.1 | (11.9) | 0.214 |
| WPPSI-R 5y | 19 | 94.8 | (17.1) | 2 | 104.0 | (9.9) | 0.470 | 87 | 107.3 | (12.3) | 14 | 95.5 | (15.3) | 0.002 |
| WISC-III 14y | 49 | 87.6 | (19.8) | 11 | 86.8 | (13.8) | 0.905 | 74 | 98.3 | (14.1) | 7 | 84.1 | (6.5) | <0.001 |
| Low estimated IQ, n (%) | 49 | 9 | (18.4) | 11 | 2 | (18.2) | 1.000 | 74 | 2 | (2.7) | 7 | 0 | (0) | 1.000 |

BPD: Bronchopulmonary dysplasia, BSID: Bayley Scales of Infant Development, IQ: Intelligence quotient, IQR: Interquartile range, IRDS: Infant respiratory distress syndrome, IVH: Intraventricular haemorrhage, NEC: Necrotizing enterocolitis, NICU: Neonatal intensive care unit, PDI: Psychomotor Development Index, PDMS: Peabody Developmental Motor Scales, MABC: Movement Assessment Battery for Children, MDI: Mental Development Index, SD: Standard deviation, SES: Socioeconomic status, VLBW: Very low birth weight, WPPSI-R: Wechsler Preschool and Primary Scale of Intelligence – Revised, WISC-III: Wechsler Intelligence Scale for Children – Third edition.

p-values for differences in continuous variables based on Student’s t-test, except for Parental SES and Days in NICU, where p-values are based on Mann-Whitney U test. p-values for differences in proportions based on Pearson’s chi square test.
