# Supplementary material for: Outcomes and predictors of functioning, mental health, and health-related quality of life in adults born with very low birth weight: a prospective longitudinal cohort study
Source: BMC Pediatr. 2022 Nov 3;22:628. doi: 10.1186/s12887-022-03676-6 (PMC9632018; doi:10.1186/s12887-022-03676-6)
Supplement: Supplementary file 2 — Supplementary Material 2 [file 12887_2022_3676_MOESM2_ESM.docx]

**Table S2.** Standardised regression coefficients for predictors of adult outcomes in very low birth weight participants.

|  | **ASR Mean Adaptive** | **GAF Function** | **GAF Symptom** | **HADS Total** | **SF-36 Mental** | **SF-36 Physical** |
| --- | --- | --- | --- | --- | --- | --- |
|  | β | β | β | β | β | β |
| **Maternal factors** |  |  |  |  |  |  |
| Maternal glucocorticoids | 0.01 | 0.11 | 0.02 | -0.13 | 0.05 | -0.03 |
| Parental SES | -0.04 | -0.09 | -0.11 | 0.18 | -0.31 | 0.32 |
| **Perinatal factors** |  |  |  |  |  |  |
| Birth weight (pr.100 g) | 0.12 | 0.38 | 0.19 | -0.34 | 0.19 | -0.12 |
| Gestational age (weeks) | 0.21 | 0.10 | 0.11 | -0.21 | 0.09 | -0.13 |
| Apgar at 5 min | 0.02 | 0.14 | 0.08 | -0.19 | 0.09 | -0.13 |
| Days with respiratory support | -0.15 | -0.39 | -0.41 | 0.41 | -0.35 | 0.17 |
| Days in NICU | 0.06 | -0.21 | -0.05 | 0.19 | -0.03 | -0.11 |
| IVH | 0.03 | -0.28 | -0.36 | 0.31 | -0.27 | 0.18 |
| BPD | -0.16 | -0.24 | -0.21 | 0.27 | -0.10 | 0.03 |
| Sepsis at birth | 0.23 | -0.01 | 0.19 | -0.11 | 0.20 | -0.09 |
| **Motor function** |  |  |  |  |  |  |
| BSID PDI 1y | 0.09 | -0.05 | -0.13 | -0.25 | 0.12 | 0.33 |
| PDMS Eye-hand 5y | 0.09 | 0.45 | 0.33 | -0.32 | 0.17 | 0.52 |
| PDMS Balance 5y | 0.27 | 0.22 | 0.14 | -0.19 | 0.08 | 0.51 |
| PDMS Locomotor 5y | 0.20 | 0.37 | 0.21 | -0.25 | 0.14 | 0.48 |
| MABC Total 14y | -0.08 | -0.40 | -0.44 | 0.55 | -0.55 | -0.21 |
| Cerebral palsy | -0.10 | -0.38 | -0.18 | 0.07 | 0.03 | -0.03 |
| **Cognitive function** |  |  |  |  |  |  |
| BSID MDI 1y | 0.06 | -0.06 | -0.29 | 0.16 | -0.34 | 0.25 |
| WPPSI 5y | -0.05 | 0.01 | -0.07 | -0.05 | -0.19 | 0.58 |
| WISC-III 14y | -0.10 | 0.41 | 0.20 | -0.16 | 0.11 | 0.31 |

ASR: Adult Self-Report, β: Standardised beta, BPD: Bronchopulmonary Dysplasia, BSID: Bayley Scales of Infant Development, IVH: Intraventricular Haemorrhage, MABC: Movement Assessment Battery for Children, MDI: Mental Development Index, PDI: Psychomotor Development Index, PDMS: Peabody Developmental Motor Scales, SES: Socioeconomic status, SF-36: Short Form 36 Health Survey, WISC-III: Wechsler Intelligence Scale for Children – Third edition, WPPSI-R: Wechsler Preschool and Primary Scale of Intelligence – Revised.

Linear regression using ASR Mean Adaptive score, GAF Function and Symptom scores, HADS Total score and SF-36 Physical and Mental Component Summaries as dependent variables, and maternal and perinatal factors as well as motor and cognitive function as independent variables.
